# Physical exercise as a modulator of the sleep–obesity interaction: metabolic, neuroendocrine and circadian mechanisms

**DOI:** 10.3389/frsle.2026.1943600

**Published:** 2026-09-11

**Authors:** Raquel M. S. Campos, Helton de Sá Souza, Carolina Machado Favaron, Guilherme Pereira Saborosa, Luciana Cervi Ferez, Sergio Tufik, Hanna Karen Moreira Antunes, Vânia D'Almeida, Marcos Mônico-Neto

**Affiliations:** 1 Programa de Pós-Graduação Interdisciplinar em Ciências da Saúde, Universidade Federal de São Paulo, Santos, Brazil; 2 Departamento de Educação Física, Universidade Federal de Viçosa, Viçosa, MG, Brazil; 3 Departamento de Psicobiologia, Universidade Federal de São Paulo, São Paulo, Brazil; 4 Instituto do Sono – Associação Fundo de Incentivo à Pesquisa (AFIP), São Paulo, SP, Brazil

**Keywords:** chronoexercise, circadian rhythms, inflammation, metabolism, obesity, obstructive sleep apnea, physical activity, sleep

## Abstract

Obesity and sleep disturbances are highly prevalent chronic conditions that share multiple pathophysiological mechanisms, including chronic low-grade inflammation, neuroendocrine dysfunction, circadian misalignment, autonomic imbalance, insulin resistance, and metabolic dysregulation. These conditions establish a multidirectional relationship that contributes to the development and progression of cardiometabolic, respiratory, and psychological comorbidities. In this context, physical exercise (PE) is widely recognized as a non-pharmacological strategy for the prevention and treatment of obesity and sleep disturbances that simultaneously modulates these interconnected pathways while enhancing metabolic flexibility. Growing evidence also supports its role as a non-photic *zeitgeber* that synchronizes central and peripheral biological clocks, modulates clock gene expression, and influences metabolic responses according to the time of day it is performed. Through these multisystem adaptations, PE may interrupt the vicious cycle linking obesity and sleep disturbances, thereby improving metabolic health and reducing the burden of obesity-related comorbidities. This review discusses how PE modulates the multidirectional relationship between sleep and obesity through integrated multisystem adaptations, highlighting its potential to improve both sleep and overall health.

## 1 Introduction

Obesity is one of the most prevalent chronic diseases today and poses a significant public health challenge due to its association with various metabolic, cardiovascular, and neurobehavioral complications (World Health Organization, 2025). Traditionally attributed to an imbalance between energy intake and expenditure, its etiology is now recognized as multifactorial, involving genetic, environmental, behavioral, and physiological aspects that interact in complex ways throughout the lifespan (Chooi et al., 2019; Blüher, 2025). Among them, sleep and biological rhythms have assumed a central role in contemporary scientific discussions. Epidemiological and experimental evidence demonstrates that short sleep duration, poor sleep quality, irregular sleep-wake schedules, and circadian misalignment are associated with an increased risk of obesity, insulin resistance, type 2 diabetes, and cardiovascular disease (Chattu et al., 2018; Lange et al., 2024). Furthermore, alterations in the circadian timing system can influence energy metabolism, appetite, glycemic homeostasis, hormone secretion, and eating behavior, thereby contributing to the development and maintenance of excess weight (Poggiogalle et al., 2018).

Sleep disorders frequently coexist with obesity and can exacerbate its adverse effects. Together with them, obstructive sleep apnea (OSA) stands out due to its high prevalence in people with obesity and its association with intermittent hypoxia, sleep fragmentation, oxidative stress, systemic inflammation, and sympathetic hyperactivity (Jordan et al., 2014; Lévy et al., 2015). However, OSA represents only one of the multiple mechanisms by which alterations in sleep and the temporal organization of biological processes can impact overall health. More broadly, the interplay between obesity, inadequate sleep, and circadian misalignment appears to contribute to a physiological environment characterized by chronic inflammation, autonomic dysfunction, metabolic alterations, and impaired quality of life (Hotamisligil, 2006; Ryan, 2017).

**Figure 1:**
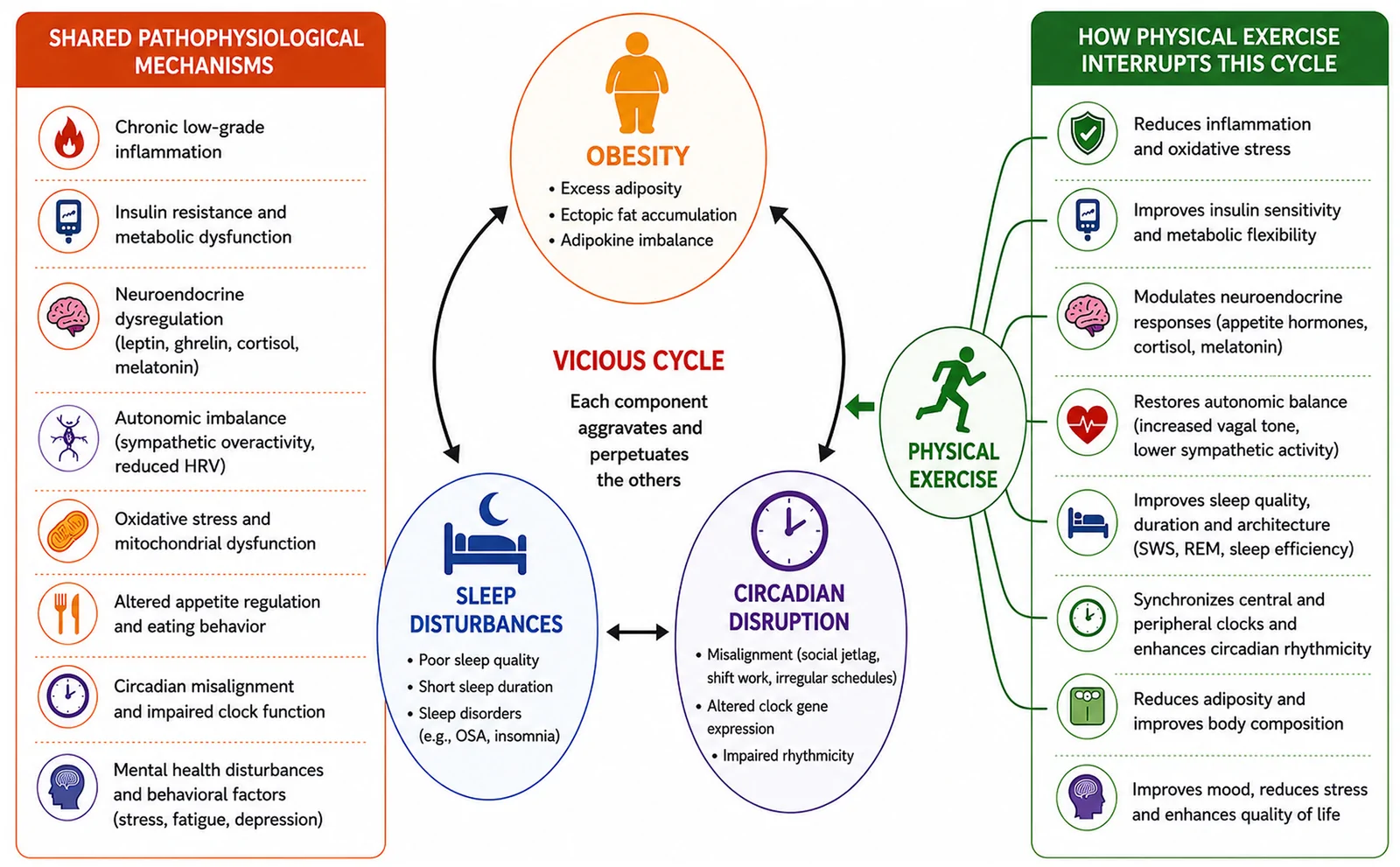
Interconnected mechanisms linking obesity, sleep disturbances, and circadian disruption, and the multiple pathways through which physical exercise may interrupt this vicious cycle. HRV, heart rate variability; SWS, slow-wave sleep; REM, rapid eye movement sleep; OSA, obstructive sleep apnea.

In this context, physical exercise (PE) has emerged as a non-pharmacological strategy capable of simultaneously acting on several of these mechanisms. In addition to its well-established effects on body composition, energy expenditure, and cardiorespiratory fitness, regular PE may improve sleep quality, facilitate circadian synchronization, reduce inflammatory and oxidative stress markers, and promote beneficial autonomic and metabolic adaptations (Hawley et al., 2014; Mendelson et al., 2018) (Figure 1).

The interaction between PE and the circadian system encompasses both molecular and behavioral mechanisms. PE can act as a non-photic *zeitgeber*, influencing peripheral clocks, clock-gene expression, body temperature, hormonal rhythms, and metabolic responses (Youngstedt et al., 2019; Ciorciari and Lamia, 2025). Conversely, circadian phase and individual chronotype may influence PE performance and physiological responses, indicating that the effects of exercise timing cannot necessarily be generalized across individuals. Indeed, current evidence does not support a universally optimal time of day for PE, and individual circadian characteristics, habitual exercise timing, and the alignment between training and performance should be considered when evaluating time-of-day effects (Bruggisser et al., 2023). This bidirectional relationship provides the basis for chronoexercise, in which PE timing is considered alongside individual circadian characteristics as a potential strategy to optimize metabolic and circadian health and, under conditions of circadian disruption, potentially facilitate circadian realignment (Youngstedt et al., 2019; Ciorciari and Lamia, 2025).

Despite advances in this field, the mechanisms by which PE simultaneously influences obesity, sleep, and the circadian system remain incompletely understood. Elucidating the interactions among these processes may contribute to the development of more integrated and effective therapeutic approaches. Accordingly, this review aims to synthesize current evidence on the relationships among obesity, sleep, biological rhythms, and PE, discuss the underlying physiological mechanisms, and highlight the potential health implications of these interactions.

### 1.1 Effects of physical exercise on weight control, neuroendocrine and inflammatory modulation

Obesity represents a major public health challenge, given its increasing prevalence and its association with a broad range of functional and metabolic complications, including musculoskeletal disorders, chronic low-grade inflammation, and insulin resistance. According to data from the World Health Organization (2025), in 2022, approximately 2.5 billion adults were living with overweight (body mass index ≥25 kg/m^2^), including more than 890 million adults with obesity (body mass index ≥30 kg/m^2^). These numbers are expected to increase substantially, with projections suggesting that by 2030, more than 2.9 billion adults could be living with overweight, including approximately 1.2 billion with obesity (World Obesity Federation, 2025). Obesity also imposes a considerable economic burden, driven by increased healthcare expenditures, morbidity, premature mortality, absenteeism, and reduced work productivity. Considering changes in population size and age structure, and assuming that current trends in obesity prevalence persist, the global economic costs associated with overweight and obesity are projected to increase from approximately US$2 trillion in 2020 to more than US$3 trillion by 2030, reaching over US$18 trillion by 2060, expressed in 2019 US dollars (Okunogbe et al., 2022; World Obesity Federation, 2022). Furthermore, analyses across 161 countries estimate that the economic costs associated with overweight and obesity could exceed 3.29% of global gross domestic product by 2060 (Okunogbe et al., 2022).

Sedentary behavior and physical inactivity are also key factors associated with the development and worsening of obesity. Physical activity (PA) encompasses any bodily movement produced by skeletal muscles that results in energy expenditure, including activities performed in occupational, domestic, transportation, and leisure contexts. PE, in turn, constitutes a subcategory of PA characterized by planned, structured, and repetitive movements performed with the purpose of maintaining or improving physical fitness, functional performance, and health (Donnelly et al., 2009; Bull et al., 2020; Oppert et al., 2021; Bishop et al., 2025). This distinction is particularly relevant in the context of obesity, as the health benefits associated with an active lifestyle extend beyond structured exercise.

Physically active individuals have a lower risk of all-cause mortality (Momma et al., 2022; Kraus et al., 2019), as well as a lower incidence of cancer, hypertension (World Health Organization, 2024), and type 2 diabetes (Momma et al., 2022). Regular PA is also associated with improvements in body composition and reductions in body fat (Donnelly et al., 2009), lower risks of depression and cognitive decline (Pearce et al., 2022), and better sleep quality (World Health Organization, 2024).

In individuals with obesity, regular PE is associated with improvements in strength and cardiorespiratory fitness (Oppert et al., 2021), increased fat oxidation and mitochondrial activity (Stroh and Stanford, 2023), enhanced insulin sensitivity and reduced liver fat (Pojednic et al., 2022), improved hunger and satiety signaling (Gómez Escribano et al., 2017), better blood pressure control (Oppert et al., 2021), and improvements in biochemical parameters, such as glycated hemoglobin, triglycerides, and HDL cholesterol, independent of weight loss (Pojednic et al., 2022).

The isolated impact of PE on weight loss may be modest, with reductions averaging 2–3 kg; however, its practice plays an important role in obesity management, particularly regarding body composition and the prevention of weight regain. It is recommended that individuals with obesity perform 150–200 min of moderate-intensity aerobic exercise per week, while higher volumes, between 200 and 300 min, may promote greater weight loss (Oppert et al., 2021).

Aerobic exercise, as well as high-intensity interval training (HIIT), has also been associated with dose-response reductions in body weight, waist circumference (Donnelly et al., 2009), body fat percentage, and visceral adipose tissue (Brennan et al., 2019; Oppert et al., 2021). This effect was observed by Berge et al. (2021), who investigated the effects of aerobic exercise intensity on energy expenditure and body composition in individuals with severe obesity undergoing an exercise training program. Participants were allocated to moderate-intensity continuous training or high-intensity training groups. In both groups, energy expenditure increased during PE and was associated with reductions in body weight, without significant changes in basal metabolic rate. However, high-intensity training yielded more substantial results across the variables analyzed. Similarly, Creasy et al. (2025)) observed that individuals participating in a weight-loss program who achieved a weekly training volume of ≥150 min showed greater reductions in body weight and body fat, as well as greater increases in aerobic capacity, compared with those who performed < 150 min of weekly PE.

Although resistance training has a lower impact on body weight reduction through increased energy expenditure compared with aerobic exercise (Willis et al., 2012), it is recommended during the weight-loss process to preserve lean mass, preferably at moderate-to-high intensity, with a minimum frequency of two sessions per week (Donnelly et al., 2009; Oppert et al., 2021). Furthermore, resistance training provides important benefits for obesity management, including increased muscle mass, which may contribute to maintaining or increasing basal metabolic rate, as well as improvements in functional capacity and muscle strength (Donnelly et al., 2009; Oppert et al., 2021).

Studies comparing combined resistance and aerobic training with single-mode training programs have reported improvements in muscle mass and strength, as well as insulin sensitivity and skeletal muscle glucose uptake (van Baak et al., 2021). Improvements in inflammatory markers and cardiorespiratory fitness have also been observed (Lopez et al., 2022), while combined exercise interventions may additionally benefit functional capacity, including flexibility, balance, and walking speed, and quality of life (Al-Mhanna et al., 2024). The effects of PE may also be enhanced when combined with other obesity treatments.

Regular PE also acts on pathways involved in energy expenditure and metabolic homeostasis and may influence the neuroendocrine control of food intake (Zhang et al., 2024; Tanwar and Kalpana, 2025). Food intake and satiety are regulated through central and peripheral signals involving different neuropeptides and hormones, including insulin, cholecystokinin (CCK), ghrelin, leptin, glucagon-like peptide-1 (GLP-1), peptide YY (PYY), alpha-melanocyte-stimulating hormone (α-MSH), neuropeptide Y (NPY), melanin-concentrating hormone (MCH), and agouti-related peptide (AgRP; Alhabeeb et al., 2021).

Obesity can alter neuroendocrine food-intake signaling, particularly through leptin resistance, which is commonly observed in individuals with obesity (Jung and Kim, 2013). PE can modulate appetite in people with obesity through physiological mechanisms that influence eating behavior and gastric motility and reduce hunger signals (Razi et al., 2025). Distinct effects on neuropeptides involved in food intake and satiety have also been observed according to the acute or chronic effects of PE.

Chronic PE appears to reduce appetite and food intake and increase energy expenditure, potentially through mechanisms involving greater anorexigenic activity, which may contribute to reductions in body weight (Ibeas et al., 2021). In adolescents with obesity, the neuroendocrine response to PE may also vary according to PE modality and intervention duration. After 1 year of an interdisciplinary intervention including regular aerobic exercise, an increase in MSH, an anorexigenic neuropeptide, was observed. However, when aerobic exercise was combined with resistance training, AgRP, an orexigenic neuropeptide, increased after 6 months and decreased after 1 year of intervention. These findings suggest that exercise modality and duration may influence neuroendocrine responses involved in energy balance regulation (Carnier et al., 2013).

Furthermore, longitudinal interventions involving regular PE in adolescents with obesity showed significant reductions in leptin and MCH, with no changes in ghrelin levels (Carnier et al., 2008, 2010). More recently, a meta-analysis demonstrated that individuals with overweight or obesity who engaged in regular PE experienced benefits including reductions in body weight and body mass index; however, across most studies involving different exercise modalities, ghrelin concentrations were increased or unchanged (Xin et al., 2025).

Regarding gastrointestinal peptides, a randomized controlled trial in healthy males undergoing different PE protocols found that PYY levels increased during and after aerobic training, whereas ghrelin levels decreased during both aerobic and resistance exercise. Subjective perceptions of hunger were also reduced during and after both exercise protocols (Broom et al., 2009). Similarly, Liu et al. (2023) investigated the effects of moderate- and low-load resistance training (85% and 45%, respectively) on appetite regulation in healthy young men. Immediately after both exercise protocols, PYY concentrations increased, whereas ghrelin concentrations and hunger perception decreased compared with the control condition.

In the same context, Anderson et al. (2024) showed that high-intensity aerobic exercise may be more effective than moderate-intensity exercise in reducing hunger perception and ghrelin concentrations, with a potential influence of sex on these responses. Regarding NPY, an orexigenic biomarker involved in the neuroendocrine control of food intake, Asri et al. (2024) reported that 60 min of cycling at 60% of peak oxygen consumption (V°O_2_peak) in men with obesity suppressed this neuropeptide and was associated with reduced appetite perceptions, although no differences were observed in leptin concentrations.

PE may also contribute to the modulation of the inflammatory state associated with obesity, particularly when combined with medical support, nutritional interventions, and psychological counseling (Dâmaso et al., 2024, 2026). Several studies have reported positive changes in adipose tissue metabolism associated with PE, with potential improvements in metabolic function and reductions in inflammation.

PE may also promote the conversion of white adipose tissue into beige adipose tissue through a process known as browning, which may contribute to increased energy expenditure (Sanchez-Delgado et al., 2015; Stroh and Stanford, 2023). In obesity, metabolic alterations in white adipose tissue, primarily driven by adipocyte hypertrophy and hypoxia, promote adipose tissue dysfunction and increased secretion of pro-inflammatory mediators, including interleukin-6 (IL-6), tumor necrosis factor-alpha (TNF-α), and leptin, accompanied by reductions in anti-inflammatory markers such as adiponectin. This condition contributes to obesity-related comorbidities and metabolic diseases (Kawai et al., 2021).

Jeong et al. (2026) showed that adolescents with obesity who underwent 12 weeks of regular plyometric exercise had improvements in body mass index and body fat mass, reductions in leptin, and increases in adiponectin levels, indicating potential anti-inflammatory and metabolic benefits. Similarly, a recent meta-analysis demonstrated positive effects of regular PE on inflammatory biomarkers and abdominal obesity in older adults, with benefits observed across different modalities, including combined, aerobic, and resistance training (Liu et al., 2026). Adiponectin is an anti-inflammatory adipokine that is generally reduced in obesity. Mallardo et al. (2023) reported that a PE protocol based on walking, running, or a combination of both in young males with obesity increased high-molecular-weight adiponectin, which is recognized for its metabolic functions.

In adolescents with obesity, reductions in the homeostasis model assessment of adiponectin (HOMA-AD) and increases in adiponectin concentrations were observed only after an interdisciplinary weight-loss protocol combining aerobic and resistance training, compared with aerobic exercise alone (da Silveira Campos et al., 2017). IL-6 levels are also higher in individuals with obesity than in non-obese populations across childhood, adolescence, and adulthood, contributing to a pro-inflammatory state and increased cardiometabolic risk. Regular PE can reduce pro-inflammatory biomarkers in obesity, including IL-6 (De Filippo et al., 2015).

A meta-analysis reported that combined aerobic and resistance training was the most beneficial PE modality for improving inflammatory markers and body composition in individuals with overweight or obesity compared with isolated aerobic exercise, resistance training, or HIIT (Wang et al., 2022). Overall, these findings support the role of regular PE in improving body composition, neuroendocrine regulation of energy balance, and the pro-inflammatory state in individuals with obesity, particularly when combined with other clinical interventions (Dâmaso et al., 2024, 2026).

The adaptive response to PE can exhibit substantial inter-individual variability and is influenced by biological, metabolic, and behavioral factors. Genotype and other individual characteristics may modulate exercise-induced physiological adaptations (Timmons et al., 2010), while training-related factors, including recovery between sessions and the ability to achieve prescribed training loads, may also influence the response (Mann et al., 2014; Pickering and Kiely, 2019). Circadian preferences, social schedules, baseline fitness levels, dietary composition, emotional state, sleep quality, body weight, and age may further contribute to this variability. Therefore, adjustments in intervention timing, training intensity and volume, exercise modality, and recovery may help promote more effective and individualized responses.

Among these factors, individual differences in circadian timing, represented by chronotype and social jetlag (SJL), may be especially relevant. Chronotype reflects an individual's preference for earlier or later sleep and activity timing (Roenneberg et al., 2012; Adan et al., 2012), whereas SJL refers to the misalignment between biological and socially imposed schedules (Wittmann et al., 2006). Both later chronotype and greater SJL have been associated with obesity and adverse metabolic outcomes (Mathew et al., 2020; Zhang et al., 2022; Arab et al., 2024; Lin et al., 2024). In addition, evening chronotypes have been associated with lower levels of PA and greater sedentary behavior (Sempere-Rubio et al., 2022), suggesting that circadian preferences may also influence engagement in regular exercise. Thus, considering circadian preferences and social schedules may contribute to a more individualized approach to exercise prescription.

Despite the various benefits associated with regular PE, individuals with obesity frequently face individual, social, and environmental barriers to initiating and maintaining a training routine. Among the main limiting factors are obesity-related comorbidities, low motivation, difficulties with adherence, pain and physical discomfort, fatigue, lack of time or competing priorities, and limited social, family, and peer support.

Environmental barriers, such as limited access to suitable facilities, neighborhood insecurity, and lack of professional guidance, can also reduce PE levels in this population. Conversely, improvements in physical fitness, physical and mental wellbeing, and perceived pleasure during PE have been associated with higher levels of PE (Baillot et al., 2021), while resilience and realistic expectations regarding outcomes may also contribute to greater engagement (Curran et al., 2023). A sense of safety and accessibility in the living environment has likewise been associated with higher levels of PE (Deslippe et al., 2023).

Taken together, these findings suggest that interventions that consider individual preferences, motivation, physical and clinical capacity, available time, environmental context, and circadian characteristics when prescribing PE may help optimize outcomes and promote long-term adherence.

### 1.2 Circadian regulation by physical exercise

Since sleep is regulated and intrinsically linked to the circadian system, the assessment of sleep effects on metabolic health and the development of obesity should also focus on circadian health. Previous studies have shown that circadian disruption is directly associated with metabolic dysfunctions, such as glucose intolerance, insulin resistance, obesity, and alterations in energy homeostasis (Poggiogalle et al., 2018; Chaput et al., 2023). Therefore, the circadian rhythm should be considered a fundamental factor in efforts to reduce the metabolic consequences of sleep debt. Given that circadian health is a fundamental aspect of metabolic regulation, interventions aimed at synchronizing and strengthening biological rhythms deserve greater attention. This perspective is particularly relevant when examining the intricate relationship between sleep disturbances and obesity. Indeed, a growing body of evidence accumulated in recent decades indicates that disruption of the circadian rhythm contributes to the development of numerous pathological conditions, many of which are closely linked to metabolic dysfunction (Bass and Takahashi, 2010; Maury, 2019).

Circadian disruption not only results from obesity but also contributes to its progression, creating a self-perpetuating cycle of metabolic dysfunction (Chaput et al., 2023). Individuals with obesity frequently exhibit altered melatonin rhythmicity, impaired peripheral clock synchronization, and disturbances in cortisol rhythmicity (Chaput et al., 2023; Cipolla-Neto and Amaral, 2018; McHill and Wright, 2017). These alterations contribute to insulin resistance, impaired glucose homeostasis, reduced metabolic flexibility, impaired lipid oxidation, and dysregulation of appetite-related hormones, including leptin and ghrelin (Qian and Scheer, 2016; Stenvers et al., 2019; Taheri et al., 2004). Consequently, appetite regulation and satiety signaling become impaired, favoring excessive energy intake, weight gain, and further disruption of circadian organization. This multidirectional interaction establishes a vicious cycle in which obesity and circadian misalignment mutually reinforce one another, thereby accelerating metabolic deterioration (Bass and Takahashi, 2010; Chaput et al., 2023).

The circadian system regulates the sleep-wake cycle and various physiological and behavioral processes. In mammals, the central biological clock is located in the suprachiasmatic nucleus of the hypothalamus, is primarily synchronized by light, and coordinates the peripheral clocks present in different tissues (Reppert and Weaver, 2002). While the central clock responds predominantly to light stimuli, peripheral clocks are influenced by non-light factors such as food intake, body temperature, hormonal signals, and also PA (Healy et al., 2021). Disorganization between central and peripheral clocks, common in situations such as shift work and frequent travel across different time zones, can lead to circadian misalignment, a condition associated with the development and worsening of various chronic diseases. Therefore, habits that support circadian health and homeostasis are essential for preventing these diseases (Healy et al., 2021).

At the molecular level, circadian rhythms are generated by a highly conserved transcriptional–translational feedback loop comprising core clock genes. The transcription factors CLOCK and BMAL1 form a heterodimer that activates the expression of the *Period* (*Per1–3*) and *Cryptochrome* (*Cry1–2*) genes. Subsequently, PER and CRY proteins accumulate in the cytoplasm, translocate to the nucleus, and inhibit CLOCK:BMAL1 activity, thereby generating self-sustained oscillations with an approximately 24-h periodicity. Additional regulatory components further stabilize this molecular clock by modulating the transcription of *Bmal1*. Beyond maintaining temporal organization, clock genes regulate the rhythmic expression of numerous downstream genes involved in glucose metabolism, insulin signaling, lipid metabolism, mitochondrial function, and energy homeostasis (Takahashi et al., 2008). Consequently, alterations in clock gene expression or function have been associated with circadian disruption and an increased risk of metabolic disorders, including obesity and type 2 diabetes (Takahashi et al., 2008).

**Figure 2:**
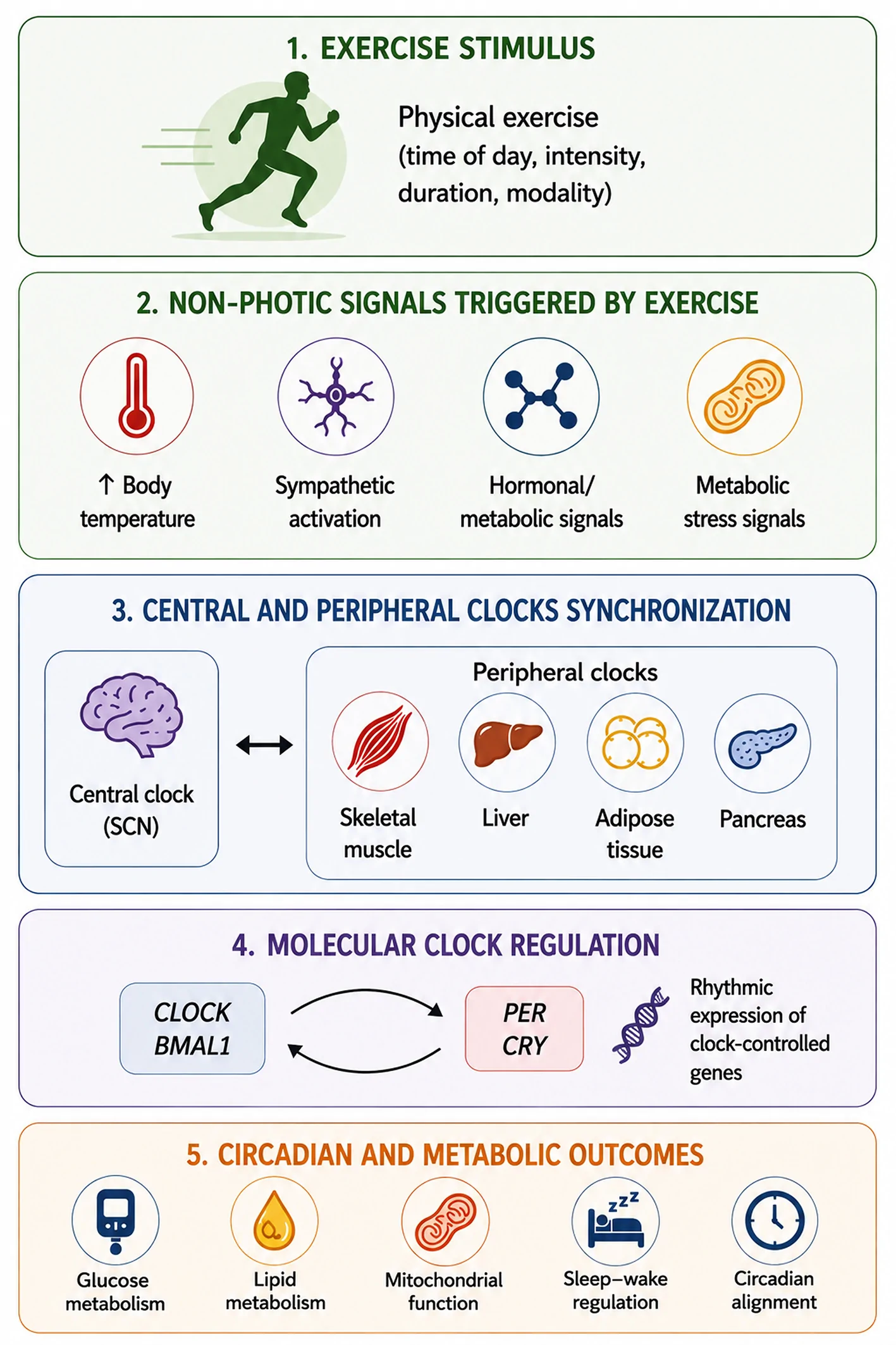
Physical exercise acts as a non-photic *zeitgeber*, influencing central and peripheral clocks and molecular clock regulation, with downstream effects on circadian and metabolic processes. SCN, suprachiasmatic nucleus.

From this perspective, PE can be considered an essential physiological modulator, capable of simultaneously influencing metabolic, neuroendocrine, and circadian processes, contributing to health and wellbeing, but also acting at the molecular level, leading to modifications in the expression of clock genes or genes controlled by the clock, as we will discuss later. Beyond their widely recognized metabolic benefits, PA and exercise also play a crucial role in chronobiology, serving as key behavioral non-photic time-givers (*Zeitgebers*) that synchronize circadian rhythms and promote phase shifts in biological rhythms, particularly in melatonin secretion (Atkinson et al., 2007). Current evidence supports the notion that regular PE can reinforce the alignment between central and peripheral biological clocks, thus improving the temporal coordination of physiological functions, including the regulation of the sleep-wake cycle, hormone secretion, and energy metabolism (Youngstedt et al., 2019; Stenvers et al., 2019). Through these mechanisms, PE contributes to greater circadian robustness and promotes more consolidated, higher-quality sleep, both of which are essential determinants of metabolic health. Although light remains the primary environmental *zeitgeber*, PE is increasingly recognized as a powerful non-photic synchronizer, helping regulate circadian rhythms and strengthening alignment among biological clocks. Consequently, in the complex interaction between sleep disorders and obesity, exercise emerges as a valuable non-pharmacological strategy to improve circadian health, support metabolic regulation, and maintain overall physiological homeostasis (Wolff and Esser, 2019; Gabriel and Zierath, 2019; Shen et al., 2023) (Figure 2).

Although structured PE has been more extensively investigated as a non-photic *zeitgeber*, it should be recognized that exercise represents only one component of the broader spectrum of PA. During wakefulness, habitual PA is accompanied by physiological activation of the sympathetic nervous system and the hypothalamic–pituitary–adrenal axis, leading to transient increases in circulating catecholamines and glucocorticoids (Healy et al., 2021). These neuroendocrine signals contribute to the entrainment of peripheral clocks by modulating the expression of core clock genes (Healy et al., 2021; Dyar et al., 2018). Experimental evidence indicates that even in the absence of an acute bout of exercise, physiological elevations in epinephrine during the wake phase are sufficient to induce *Per1* expression and restore dampened oscillations of *Bmal1* and *Per2* in peripheral tissues, demonstrating that circadian entrainment is not exclusive to physical training (Terazono et al., 2003). Because structured exercise elicits a more robust sympathetic and hormonal response, its synchronizing effects are likely to be greater, suggesting that the circadian adaptations induced by movement occur along a dose-dependent continuum according to the magnitude of the physiological stimulus (Goldsmith and Bell-Pedersen, 2013; Healy et al., 2021).

When evaluating these relationships, it is also relevant to consider age-related factors, as PE has consistently been shown to be an effective strategy for preventing obesity in children and adolescents. Thus, the World Health Organization (2024), in its recent guidelines, emphasizes that children and adolescents should achieve adequate sleep in both duration and quality, reinforcing the critical role of biological rhythms in obesity prevention across these age groups (Dou et al., 2025).

Although considerable evidence exists regarding the role of PE as a non-photic synchronizer of the circadian system, its ability to modulate biological rhythms appears to depend on characteristics such as the timing, intensity, and duration of exercise (Atkinson et al., 2007; Shen et al., 2023). Among these factors, the time of day when exercise is performed plays a particularly relevant role. Available data indicate that exercise during the night and early morning hours is more consistently associated with delays in circadian phase, whereas the effects of daytime exercise are less well-established (Atkinson et al., 2007; Shen et al., 2023). Taken together, these findings illustrate the complexity of exercise-induced circadian responses and reinforce the need for further studies to clarify how PE can be optimally prescribed to improve circadian alignment.

Importantly, the optimal timing of PE may also depend on individual circadian characteristics, including chronotype and SJL. Later chronotype and greater SJL have been associated with obesity and adverse metabolic outcomes (Zhang et al., 2022; Arab et al., 2024; Lin et al., 2024). Therefore, considering individual circadian preferences and social schedules may be relevant when determining the timing of PE interventions.

**Figure 3:**
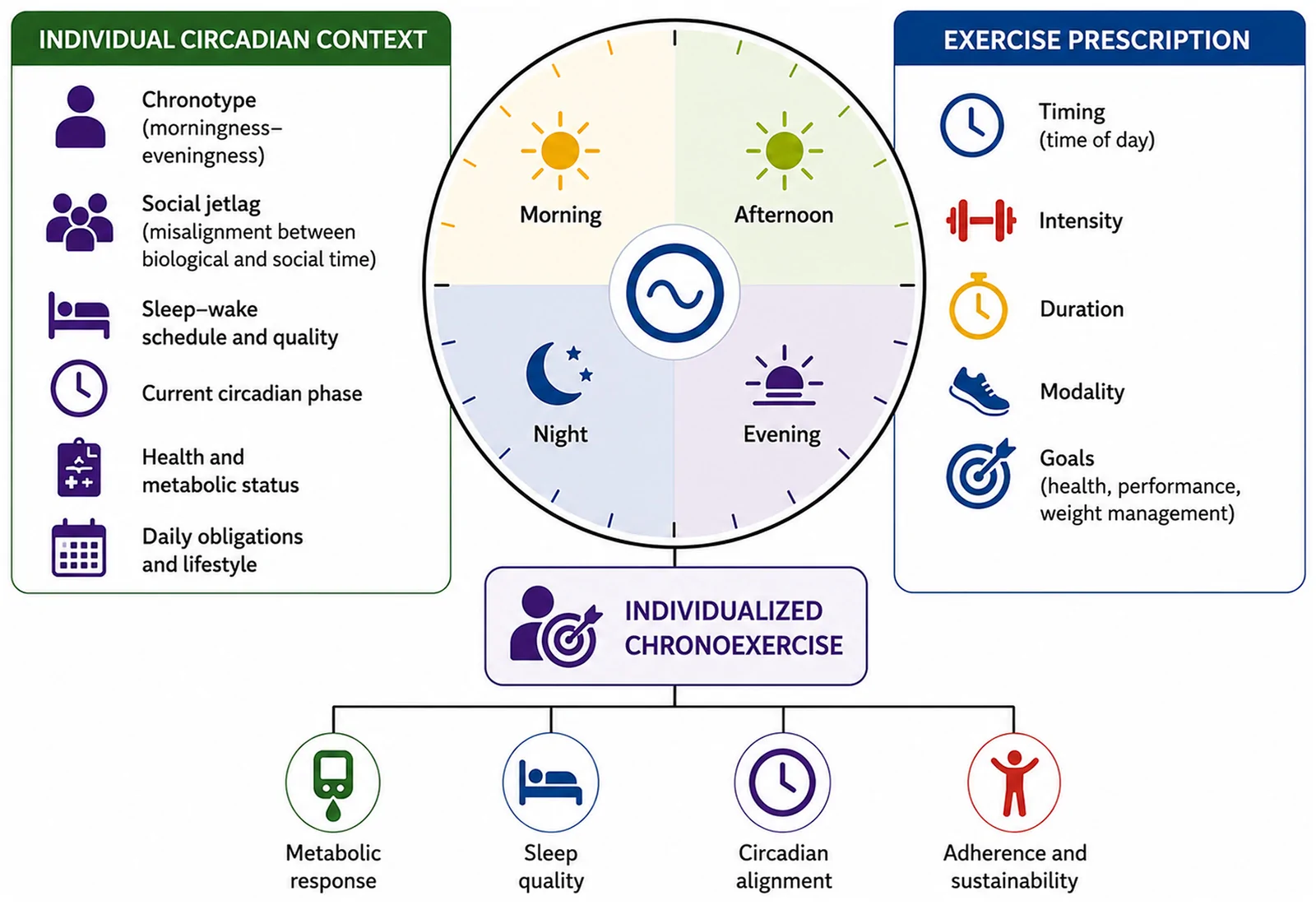
Chronoexercise integrates individual circadian characteristics with exercise timing and prescription to optimize metabolic responses, sleep, circadian alignment, and adherence.

In a recent review, Kim et al. (2023) reinforce the concept of chronoexercise, highlighting that the effects of PE on metabolic health depend not only on its intensity, duration, and frequency but also on the time of day it is performed. This emerging concept suggests that PE timing should be considered not only in relation to external clock time but also in relation to endogenous circadian phase and individual chronotype (Martin et al., 2023) (Figure 3). The authors emphasize that PE acts as a potent physiological modulator, promoting adaptations in multiple organs—especially skeletal muscle, liver, adipose tissue, and pancreas—through complex interorgan communication mediated by molecules such as myokines and hormones (Kim et al., 2023). Thus, as previously discussed, the regulation of circadian rhythms in fundamental functions, such as energy metabolism, hormone secretion, and the sleep–wake cycle, can be influenced by PE, with important implications for metabolic health.

Although studies have demonstrated associations between SJL, lower PA levels, and obesity, evidence regarding whether PE can attenuate the metabolic alterations induced by SJL remains limited, particularly in humans. Experimental evidence from animal models, however, provides preliminary support for a potential protective role of PE. In a mouse model of SJL, voluntary wheel running accelerated the resynchronization of locomotor activity and facilitated the adaptation of peripheral circadian rhythms following repeated phase shifts (Oneda et al., 2022). More recently, Dial et al. (2024) demonstrated that SJL impaired glucose homeostasis and attenuated exercise-induced metabolic and skeletal muscle adaptations, whereas voluntary exercise partially restored glucose regulation and prevented excess weight gain. Although these findings cannot yet be directly extrapolated to humans, they suggest that PE may both facilitate circadian re-entrainment and mitigate some of the metabolic consequences associated with SJL.

One of the key aspects underlying the interaction between PE and circadian regulation is the marked diurnal variation in metabolic function. Glucose tolerance, insulin sensitivity, and β-cell responsiveness are generally higher in the morning and progressively decline throughout the day, reaching their lowest levels in the evening and at night (Morris et al., 2015; Qian and Scheer, 2016). Likewise, lipid metabolism is under circadian regulation, with genes involved in lipid synthesis and storage exhibiting rhythmic expression patterns that favor triglyceride and cholesterol synthesis during the biological night (Panda, 2016; Stenvers et al., 2019). These temporal variations indicate that metabolic responses to PE may differ depending on when it is performed.

Supporting these findings, growing evidence suggests that the timing of PE can influence the magnitude of metabolic adaptations. Several studies have reported that PE performed in the late afternoon or early evening is associated with greater improvements in glycemic control, insulin sensitivity, blood glucose regulation, and lipid metabolism compared with morning PE, particularly in individuals with overweight, obesity, or type 2 diabetes (Savikj et al., 2019; Gabriel and Zierath, 2019; Teo et al., 2020). Furthermore, neuromuscular performance, including muscle strength and power output, typically peaks later in the day, which may enhance PE effectiveness (Chtourou and Souissi, 2012). Conversely, morning PE may provide specific metabolic advantages, such as increased fat oxidation during fasted conditions and a lower risk of delayed post-exercise hypoglycemia in individuals with type 1 diabetes (Van Proeyen et al., 2010; Campbell et al., 2015).

The physiological basis underlying these diurnal differences in PE responsiveness is increasingly attributed to the interaction between PE stimuli and the intrinsic molecular clock of skeletal muscle, which temporally regulates metabolic pathways and cellular function. As an example, using a tamoxifen-inducible skeletal muscle-specific *Bmal1* knockout model, Hodge et al. (2015) demonstrated that BMAL1-dependent circadian regulation coordinates the temporal expression of metabolic genes involved in glucose utilization, lipid metabolism, and muscle fiber-type specification, thereby orchestrating substrate metabolism in skeletal muscle. The intrinsic skeletal muscle clock, centered on BMAL1-dependent transcriptional regulation, plays a critical role in coordinating glucose metabolism and maintaining the temporal organization of muscle physiology. This circadian regulation provides a biological rationale for the growing interest in whether PE performed at different times of the day elicits distinct physiological and metabolic responses.

Evidence from animal models further supports the role of circadian mechanisms in exercise responsiveness. Studies in mice have demonstrated time-of-day-dependent differences in PE capacity and training adaptations, suggesting that the interaction between the molecular clock and PE stimuli may influence both performance and metabolic adaptation (Martin et al., 2023). Experimental studies have also demonstrated that scheduled PE can exert synchronizing effects on the circadian system, providing additional evidence for PE as a non-photic *zeitgeber* (Ciorciari et al., 2025). Importantly, these experimental models allow the contribution of specific clock components to exercise responses to be investigated and provide mechanistic evidence that complements findings from human studies. However, additional well-designed human studies are still needed to determine the extent and clinical relevance of the circadian synchronizing effects of PE.

An additional molecular link between circadian regulation and PE adaptation involves the cryptochrome proteins CRY1 and CRY2 and the nuclear receptor PPARδ. Jordan et al. (2017) demonstrated in mice that CRY1/2 act as co-repressors of PPARδ in skeletal muscle, limiting the expression of PPARδ target genes involved in lipid utilization and PE metabolism. Genetic deletion of Cry1 and Cry2 increased PPARδ activity and enhanced sprint PE capacity, providing direct experimental evidence that components of the circadian clock can modulate PE performance through metabolic transcriptional pathways.

Supporting the importance of PE timing, Shen et al. (2025) investigated the effects of a 12-week aerobic exercise intervention performed in the morning or evening on a range of metabolic and vascular parameters. The study demonstrated that aerobic training significantly improves physical health in sedentary young men (22–25 years). Notably, morning exercise (6–8 a.m.) was associated with greater reductions in body fat, improvements in lipid profile, and advancement of the circadian phase, while evening exercise (6–8 p.m.) conferred more pronounced benefits in vascular function. Although these findings are limited to a specific population subgroup, they highlight the importance of PE scheduling as a modifiable factor in optimizing cardiometabolic health interventions.

The role of PE, particularly its timing, is also highly relevant to muscle health. PE is a potent modulator of skeletal muscle metabolism, leading to substantial health benefits. Given that muscle metabolism exhibits a well-characterized circadian rhythm, it is reasonable to propose that the timing of PE may be a valuable therapeutic strategy to maximize its physiological benefits (Gabriel and Zierath, 2019).

Skeletal muscle possesses a highly organized intrinsic circadian system, characterized by an extensive network of genes controlled by the biological clock that regulate key processes involved in energy metabolism, mitochondrial function, substrate utilization, and muscle adaptation. Alterations in the molecular mechanisms of the skeletal muscle timing system, whether due to circadian misalignment, sleep disorders, or irregular behavioral patterns, have been associated with metabolic impairments in various chronic diseases. Several physiological functions of skeletal muscle exhibit marked diurnal variations. Late afternoon and early evening are when physical performance, muscle strength, and oxidative capacity peak. At the same time, signaling pathways related to cellular energy and metabolic stress responses tend to be more active in the morning. These findings corroborate that the metabolic and functional responses to PE can vary with the time of day at which PE is performed (Dyar et al., 2018; Gabriel and Zierath, 2019).

As a significant non-photic synchronizer, PE has also been the subject of studies on its role in the circadian molecular machinery. Experimental studies indicate that PA, in addition to inducing phase shifts in peripheral clocks, can modulate the expression of central clock genes that regulate metabolic and physiological processes. Through these mechanisms, PE contributes to circadian realignment and may help mitigate some of the adverse effects associated with sleep disorders and circadian misalignment (Dyar et al., 2018; Gabriel and Zierath, 2019).

Therefore, it is noteworthy to emphasize that the circadian effects of PE—which may depend not only on its timing but also on its modality—have been shown to interact with molecular pathways of the biological clock through distinct mechanisms, leading to specific physiological and metabolic adaptations. Thus, personalizing PE prescriptions according to individual circadian characteristics, including chronotype, as well as therapeutic goals, may help optimize the timing and effectiveness of lifestyle interventions aimed at preventing or controlling metabolic disorders. This concept has contributed to the growing interest in chronoexercise as a promising component of precision medicine approaches (Gabriel and Zierath, 2019; Wolff and Esser, 2019).

### 1.3 Influence of physical exercise on sleep architecture and quality

The multidimensional aspects of sleep, such as efficiency, latency, duration, and fragmentation, can be influenced by various factors, including the accelerated pace of contemporary life, stress, and poor lifestyle habits, such as an unbalanced diet and sedentary behavior (Nelson et al., 2022). These factors are directly associated with an increased prevalence of sleep disorders, which compromise sleep architecture and adversely affect overall health (Kovacevic et al., 2018).

Given this scenario, various approaches have been proposed for the management of sleep disorders, including pharmacological and non-pharmacological strategies (Wang et al., 2025). In this context, regular PE has been increasingly recognized as an effective intervention, not only for treating sleep disorders but also for modulating sleep architecture and its functional outcomes, including reducing the risk of obesity (Yang et al., 2012). These benefits have been consistently observed across different exercise modalities, particularly aerobic and resistance training, suggesting that the sleep-promoting effects of PE are not restricted to a single type of exercise (De Nys et al., 2022; Xu et al., 2024).

Numerous studies demonstrate that aerobic exercise promotes improvements in sleep quality in older adults, adults and adolescents of both sexes, including reductions in wake time and the number of nocturnal awakenings (Wang and Youngstedt, 2014), as well as increased sleep duration and reduced latency and occurrence of sleep disturbances (Yang et al., 2012; Abd El-Kader and Al-Jiffri, 2019), with moderate intensity frequently identified as the most effective in this context. Additionally, resistance training-based interventions have also shown consistent positive effects, with significant improvements in sleep quality across diverse populations (D'Aurea et al., 2019; Gupta et al., 2022).

Beyond improving body composition and cardiovascular responses, a notable benefit of PE is its capacity to modulate the autonomic nervous system by increasing parasympathetic activity and reducing excessive sympathetic activity (Wang et al., 2025). Heart rate variability (HRV) is a non-invasive marker of cardiac autonomic modulation that provides valuable information on physiological adaptations to PE and their relationship with sleep. Regular PE may increase HRV indices associated with vagal activity, whereas sleep deprivation has been associated with reduced parasympathetic modulation and lower HRV (Amekran and El Hangouche, 2024; Zhang et al., 2025). In addition, autonomic dysregulation may contribute to impaired sleep quality, highlighting the close relationship between sleep and autonomic function (Correia et al., 2023). Taken together, these findings suggest that HRV may represent a potential physiological link between exercise and sleep through autonomic modulation. However, HRV is also sensitive to other factors, including psychological stress, which may alter parasympathetic activity and, in turn, influence sleep quality (Thielmann et al., 2021).

Exercise-induced thermoregulation may also play a central role in this process, as the elevation in body temperature during PE may interact with the physiological decline in core body temperature that occurs during the evening and early night, a process that is important for sleep initiation and may consequently contribute to reduced sleep latency (Xie et al., 2024). Additionally, alterations in the concentrations and actions of hormones and neurotransmitters, including cortisol and catecholamines, as well as in immune system function and changes in brain electrophysiological activity, represent physiological responses to PE that modulate sleep architecture and, consequently, sleep quality (Chennaoui et al., 2015).

In light of these mechanisms, the physiological responses induced by PE directly influence sleep architecture, with one of the most consistent findings being an increase in slow-wave sleep (SWS). This outcome is widely documented in the literature, with meta-analytic evidence indicating that PE significantly increases in SWS (MD = 2.19; 95%CI = 0.35–4.03; Wang et al., 2025). However, the effects on rapid eye movement (REM) sleep are less consistent, showing a tendency toward its reduction, particularly in both acute and chronic intervention protocols (Chennaoui et al., 2015). These outcomes are also corroborated by observational studies, such as the one by Brand et al. (2010) which demonstrated that adolescents with higher weekly PA levels exhibit greater sleep efficiency, increased SWS, and reduced REM sleep.

The influence of PE on lighter sleep stages, such as N1 and N2, remains less consistent in the literature than its effects on SWS. However, recent evidence indicates that PE can modulate sleep microarchitecture within these stages. Studies demonstrate alterations in electroencephalographic activity, including increased power in the alpha and theta bands during N1 and N2, as well as increased beta activity in N2, suggesting greater stability and neural organization of sleep, possibly related to sleep spindle activity (Perrier et al., 2024). Furthermore, PE has been associated with increased total EEG power during NREM sleep, reflecting greater synchronization of brain activity and indicating improved functional quality of sleep architecture across both light and deep stages (Cassim et al., 2022).

Another relevant aspect pertains to the timing of PE. Although evidence suggests that sessions performed between 4 and 8 h before bedtime are more effective at increasing SWS, those conducted too close to bedtime (less than 4 h) may be detrimental (Leota et al., 2025). This effect can be partially explained by the thermoregulatory response, in which the elevation in body temperature during PE, followed by its subsequent reduction, increases the propensity for sleep onset and facilitates progression into deeper stages of sleep. However, the magnitude of these effects may vary with individual physical fitness levels, PE modality, and methodological differences across studies (Xie et al., 2024).

In the context of alterations in sleep architecture and quality associated with comorbidities such as obesity, PE plays a relevant role not only through its direct effects on sleep but also by attenuating the pathophysiological mechanisms linking excess adiposity to inadequate sleep (Silva et al., 2019). Reductions in body weight are among its primary outcomes and are associated with decreased systemic inflammation and improved insulin sensitivity—processes that directly influence sleep regulation (Mendelson et al., 2016). Furthermore, such adaptations may occur, at least in part, independently of substantial changes in body composition. Recent meta-analyses demonstrate that PE plays a fundamental role in improving sleep disorders in populations with obesity. Within this context, different modalities—including aerobic, resistance, and combined training—prove effective in promoting improvements in both sleep parameters and physical outcomes (Wang et al., 2026). Although obesity is not the central focus of this section, its interaction with sleep reinforces PE's role as a fundamental systemic modulator.

In summary, PE represents an effective strategy for modulating sleep architecture and quality through multiple interrelated exercise-induced physiological mechanisms. Its effects extend beyond obesity, contributing broadly to sleep regulation and health promotion. However, the magnitude of these effects may vary with intensity, exercise modality, and individual characteristics.

### 1.4 Impact of physical exercise on sleep-related respiratory symptoms

Obesity is one of the main risk factors for sleep-related breathing disorders, particularly OSA. This association is driven by anatomical and physiological alterations that increase upper airway collapsibility during sleep. Excessive adipose tissue accumulation in the cervical and abdominal regions contributes to a reduced pharyngeal lumen and impaired respiratory mechanics, making individuals more susceptible not only to intermittent hypoxia but also to the consequences of sleep fragmentation (Jordan et al., 2014). However, the interaction between obesity and OSA extends beyond excess adiposity. Although they represent distinct conditions, obesity and OSA share common pathophysiological mechanisms, including chronic low-grade inflammation, oxidative stress, autonomic dysfunction, and metabolic dysregulation, which may interact to increase the risk of cardiometabolic comorbidities (Lévy et al., 2015).

Weight loss represents an important therapeutic strategy for improving OSA severity, particularly among individuals with obesity. Clinical trials based on lifestyle interventions have demonstrated that reductions in body weight are associated with decreases in the apnea–hypopnea index (AHI) and improvements in sleep-related symptoms, reinforcing the close relationship between adiposity and upper airway collapsibility (Araghi et al., 2013a). In this context, Tuomilehto et al. (2009) demonstrated that a lifestyle intervention focused on weight reduction effectively attenuated OSA severity in overweight individuals. Specifically, each 5-kg reduction in body weight was associated with an approximate 2-event/h decrease in the apnea–hypopnea index (AHI), highlighting the importance of body weight management in the treatment of sleep-related breathing disorders (Tuomilehto et al., 2009).

The pathophysiological interaction between obesity and OSA cannot be explained solely by excess body weight or upper airway anatomy. Rather, both conditions converge on common biological pathways in which oxidative stress emerges as a central mechanism linking metabolic dysfunction to respiratory abnormalities (Lévy et al., 2015). In obesity, adipocyte hypertrophy leads to local adipose tissue hypoxia and mitochondrial dysfunction, resulting in excessive production of reactive oxygen species (ROS) and the establishment of chronic low-grade inflammation (Trayhurn et al., 2008). In OSA, repetitive cycles of intermittent hypoxia further amplify ROS generation (Lavie, 2003). Together, these alterations establish a pro-oxidative environment that activates hypoxia- and redox-sensitive signaling pathways, particularly HIF-1α and NF-κB. Collectively, these responses promote macrophage infiltration into adipose tissue, increase the production of pro-inflammatory adipokines, and induce insulin resistance, establishing a self-perpetuating cycle in which chronic inflammation and oxidative stress reinforce one another (Hotamisligil, 2006).

Activation of HIF-1α and NF-κB contributes to adipocyte dysfunction, stimulates lipolysis, and increases the release of free fatty acids (FFAs) into the circulation (Lee et al., 2014; Poulain et al., 2014). Persistently elevated circulating FFAs promote ectopic lipid accumulation in insulin-sensitive tissues, such as skeletal muscle and the liver, which contributes to systemic insulin resistance (Hotamisligil, 2006). Thus, insulin resistance is considered one of the earliest hallmarks of metabolic dysfunction associated with obesity and OSA, often preceding the development of metabolic dysfunction-associated nonalcoholic fatty liver disease (NAFLD), type 2 diabetes mellitus, and other cardiometabolic disorders (Ryan, 2017).

Beyond metabolic dysfunction, the oxidative stress and inflammatory milieu generated by obesity and OSA also exert important effects on respiratory control. Intermittent hypoxia induces adaptive changes within the carotid body through oxidative stress-dependent mechanisms, increasing peripheral chemoreceptor sensitivity (Peng et al., 2009; Lavie, 2015). Consequently, chronic exposure to intermittent hypoxia causes relatively small reductions in arterial oxygen tension (PaO_2_) to elicit exaggerated ventilatory responses, leading to transient hyperventilation following upper airway reopening. In susceptible individuals, this hyperventilation may reduce arterial carbon dioxide tension (PaCO_2_) below the apneic threshold, temporarily suppressing respiratory drive, further destabilizing ventilation. In addition to promoting ventilatory instability, enhanced peripheral chemoreceptor sensitivity also results in sustained sympathetic activation through recurrent carotid body stimulation (Peng et al., 2003). Of particular relevance, this sympathetic overactivity is not restricted to sleep but persists during wakefulness, contributing to endothelial dysfunction, hypertension, cardiac arrhythmia, and increased cardiovascular risk in patients with OSA (Ryan, 2018).

Given the pivotal roles of peripheral chemoreceptor sensitization and sympathetic overactivity in OSA pathophysiology, PE has emerged as a promising non-pharmacological strategy to improve autonomic regulation through mechanisms that extend beyond weight loss alone (Aiello et al., 2016; Guerra et al., 2019). In a randomized controlled trial, Guerra et al. (2019) implemented a six-month supervised concurrent PE program consisting of moderate-intensity aerobic and resistance training performed three times per week. The intervention significantly improved metaboreflex control and attenuated the exaggerated sympathetic responses elicited during its activation, suggesting a partial restoration of autonomic regulation in patients with OSA (Guerra et al., 2019). Similarly, Maki-Nunes et al. (2015) combined a similar supervised PE program with a hypocaloric dietary intervention over 4 months. They observed significant reductions in resting muscle sympathetic nerve activity, accompanied by improvements in peripheral and central chemoreflex sensitivity (Maki-Nunes et al., 2015). Although the latter intervention also included dietary restriction, the findings from both studies suggest that structured PE, either alone or as part of a comprehensive lifestyle intervention, can attenuate peripheral chemoreflex sensitization and sympathetic overactivity, thus partially reversing the autonomic maladaptations induced by chronic intermittent hypoxia.

These findings are further supported by studies evaluating indirect markers of autonomic function. For example, Lins-Filho et al. (2024) reported improvements in blood pressure following high-intensity interval training in patients with OSA (Lins-Filho et al., 2024), whereas Kline et al. (2013) demonstrated enhanced heart rate recovery after exercise, suggesting improved cardiac autonomic regulation (Kline et al., 2013). Collectively, these observations reinforce the concept that PE favorably modulates autonomic function in OSA through multiple physiological pathways. Nevertheless, despite these consistent improvements in intermediate cardiovascular outcomes, current evidence remains insufficient to determine whether exercise training reduces the long-term risk of hypertension, major cardiovascular events, or cardiovascular mortality in patients with OSA (Iftikhar et al., 2014; Mendelson et al., 2018). Importantly, the biological mechanisms underlying these beneficial adaptations remain incompletely understood. Although experimental studies have established oxidative stress as a central mediator of carotid body sensitization during chronic intermittent hypoxia through pathways involving ROS, HIF-1α, NADPH oxidase, endothelin-1, and pro-inflammatory signaling, there is a scarcity of animal studies investigating whether exercise training directly modulates these molecular pathways or reverses the oxidative and inflammatory alterations induced by intermittent hypoxia (Nanduri et al., 2008; Peng et al., 2009).

Another mechanism through which PE may attenuate OSA severity involves body fluid dynamics. During prolonged sitting or standing, gravitational forces promote fluid accumulation in the lower limbs. Upon assuming the supine position during sleep, part of this fluid is redistributed rostrally toward the neck, increasing peripharyngeal tissue volume and upper airway collapsibility (Redolfi et al., 2009; White and Bradley, 2013). Redolfi et al. demonstrated that 1 week of moderate-intensity walking (45 min twice daily) increased daily PA by approximately 80%, reduced overnight rostral fluid shift by 40%, decreased nocturnal neck circumference enlargement by 64%, and attenuated the reduction in pharyngeal air volume during sleep. These adaptations were accompanied by a 30% reduction in the AHI, without changes in body weight. Furthermore, the reduction in overnight rostral fluid shift explained approximately 63% of the improvement in OSA severity, highlighting lower-limb fluid redistribution as an important weight-independent mechanism by which exercise may reduce upper airway collapsibility during sleep (Redolfi et al., 2015).

PE also promotes several metabolic adaptations that may mitigate the cardiometabolic consequences of OSA. By enhancing insulin sensitivity, improving mitochondrial function, increasing lipid oxidation, and attenuating chronic low-grade inflammation, regular PE can counteract many of the metabolic disturbances associated with obesity and OSA (Hawley et al., 2014). Despite this strong biological rationale, clinical studies evaluating the effects of structured exercise programs on metabolic outcomes in patients with OSA remain relatively scarce.

Clinical intervention studies consistently suggest that structured PE programs improve metabolic health in patients with OSA. Maki-Nunes et al. (2015) demonstrated that a lifestyle intervention combining supervised exercise training with a hypocaloric diet improved components of the metabolic syndrome while reducing OSA severity. Likewise, Desplan et al. (2014) observed favorable changes in fasting glucose, body composition, blood pressure, and sleep-disordered breathing following a multidisciplinary rehabilitation program that incorporated exercise training. Consistent with these findings, a randomized clinical trial involving patients with OSA and type 2 diabetes reported improvements in postprandial glucose, insulin levels, lipid profile, and exercise capacity after aerobic exercise, with even greater benefits when exercise was combined with CPAP therapy (Shen et al., 2019).

Complementing the evidence from intervention studies, a prospective cohort study conducted by our group demonstrated that physically active individuals with OSA had a significantly lower risk of developing type 2 diabetes after an 8-year follow-up than their physically inactive counterparts. Together, these findings support the role of physical exercise as a valuable adjunctive strategy for improving cardiometabolic health in patients with OSA (Mônico-Neto et al., 2018). Nevertheless, the limited number of available studies highlights the need for well-designed randomized clinical trials to establish the independent effects of PE on metabolic dysfunction and to clarify the biological mechanisms underlying these adaptations.

Taken together, these findings indicate that the therapeutic effects of PE in OSA cannot be explained solely by weight loss. Instead, accumulating evidence suggests that regular PE simultaneously modulates multiple pathophysiological pathways, including oxidative stress, chronic inflammation, autonomic dysfunction, body fluid redistribution, and metabolic impairment. This multimodal action provides a plausible biological explanation for the broad clinical benefits of PE observed in patients with OSA, even when reductions in body weight or AHI are modest. Nevertheless, further mechanistic studies and randomized clinical trials are still needed to clarify the relative contribution of these pathways and to determine whether these physiological adaptations ultimately translate into reductions in cardiovascular morbidity and mortality.

### 1.5 Behavioral and psychological effects resulting from physical exercise

Recent advances in neuroscience, endocrinology, and psychiatry have demonstrated that excess adiposity induces significant neurobiological alterations that modify brain circuits involved in regulating mood and eating behavior (Bremner et al., 2020; Debski et al., 2024; Selman et al., 2025). Conversely, depressive and anxiety disorders may also contribute to weight gain through hormonal alterations, reduced PA, changes in eating behavior, the use of psychotropic medications, and impaired sleep quality, consequently establishing a reciprocal and self-perpetuating relationship (Raman et al., 2013; Stunkard et al., 2003; Monsalve et al., 2025).

The meta-analysis conducted by Luppino et al. (2010) demonstrated that individuals with obesity are at increased risk of developing depressive disorders over time. In contrast, individuals with depression are more likely to develop obesity, confirming the multidirectional nature of this relationship. These findings have been reinforced by subsequent reviews (Milaneschi et al., 2019; Silva et al., 2020; Jitte et al., 2024), which showed that this association remains significant even after adjusting for sociodemographic factors, lifestyle, and the presence of chronic diseases. This suggests that the relationship cannot be explained solely by confounding factors but rather reflects shared pathophysiological mechanisms.

In addition to depression, anxiety disorders are also highly prevalent among individuals with obesity. Although the strength of this association varies across studies, evidence consistently indicates a higher prevalence of generalized anxiety disorder, panic disorder, social anxiety disorder, and subclinical anxiety symptoms in individuals with overweight or obesity (Atlantis et al., 2009; Jantaratnotai et al., 2017; Amiri and Behnezhad, 2019). Anxiety also plays a significant role in eating behavior by promoting emotional eating, binge eating, and a preference for highly palatable foods rich in sugar and fat. Furthermore, the weight gain associated with these behaviors may exacerbate feelings of guilt, shame, worthlessness, body dissatisfaction, and low self-esteem, leading to the perpetuation of anxiety symptoms (Bremner et al., 2020; Dakanalis et al., 2023; Swainson et al., 2023).

Chronotype and SJL, which were previously mentioned, represent additional factors linking circadian regulation to PA/PE, psychological wellbeing, and metabolic health. Regarding chronotype, evening-types have been associated with lower levels of PA and greater sedentary behavior, suggesting that circadian preference may influence engagement in health-related behaviors (Sempere-Rubio et al., 2022; Back et al., 2023). Evening-types may also be more likely to experience reduced sleep quality and duration, particularly on weekdays, whereas these differences can become less pronounced or even disappear on weekends (Vitale et al., 2015).

The discrepancy between sleep timing on weekdays and weekends, referred to as SJL and discussed earlier in this review, has also been associated with adiposity-related outcomes, including higher body mass index, body fat, and waist circumference, although the predominantly observational evidence does not establish a causal relationship (Parsons et al., 2015; Arab et al., 2024). Importantly, its potential implications extend to mental health, as greater SJL and evening preference have been associated with poorer mental health outcomes, particularly depressive symptoms. These relationships are likely bidirectional, since psychological distress, reduced PA, and irregular sleep may, in turn, contribute to circadian disruption and maladaptive health behaviors (Baron and Reid, 2014; Meyer et al., 2024).

Circadian timing may also influence eating behavior and the temporal distribution of food intake. In addition to dietary quantity and quality, meal timing and regularity are increasingly recognized as relevant determinants of metabolic health (Reytor-González et al., 2025; Schimenes et al., 2026). Food intake also acts as a temporal cue (*zeitgeber*) for peripheral circadian clocks, interacting with pathways involved in glucose metabolism, energy balance, and appetite. Accordingly, later or irregular meal timing has been associated with obesity and adverse metabolic outcomes (Liu et al., 2024; Rovira-Llopis et al., 2024). Importantly, emerging evidence suggests that the implications of meal timing may also extend to psychological health. Greater irregularity in eating schedules, including meal timing jetlag, has been associated with depressive and anxiety symptoms, while other chrononutrition behaviors, such as breakfast skipping, have also been linked to poorer mental health outcomes (Tae and Chae, 2026; Fond et al., 2026; Tan et al., 2025). These findings suggest that the timing and regularity of food intake may represent an additional behavioral pathway linking circadian disruption to both metabolic and psychological health.

Disruptions of sleep and circadian rhythms have also been associated with adverse mental health outcomes, with shift work providing a relevant example of chronic circadian misalignment. Shift workers frequently experience sleep disturbances and have an increased risk of psychological symptoms, depressive symptoms (Torquati et al., 2019). In a large cohort study using UK Biobank data, shift work was associated with a 22% higher risk of depression and a 16% higher risk of anxiety (Xu et al., 2023). These associations may reflect the combined effects of circadian misalignment, sleep disruption, and behavioral changes accompanying shift work (Lotti et al., 2025). At the biological level, circadian disruption may also affect clock-gene expression in neural regions involved in mood regulation, potentially altering neurotransmitter signaling and increasing susceptibility to emotional disturbances (McClung, 2007).

Dysregulated eating behaviors represent another relevant component of the interplay between obesity, circadian regulation, and mental health. Binge-eating disorder (BED) is particularly important in this context because it frequently co-occurs with mood and anxiety disorders, illustrating the complex interplay between eating behavior and mental health (Kowalewska et al., 2024). Binge-eating behavior may also exhibit a temporal pattern, with binge episodes, food cravings, and increased food intake occurring more frequently later in the day. Evening chronotype has similarly been associated with binge-eating behavior, although current evidence regarding the direction and mechanisms underlying this relationship remains limited (Romo-Nava et al., 2022; Cera et al., 2026).

The temporal dimension of eating behavior further supports the relevance of considering not only what and how much individuals eat, but also when and how regularly they eat. As discussed earlier, meal timing is closely linked to circadian regulation and may provide an additional pathway through which eating behavior interacts with metabolic and psychological health (Reytor-González et al., 2025). Thus, irregular or delayed eating patterns may coexist with other manifestations of circadian disruption and contribute to a broader behavioral context associated with both obesity and poorer mental health.

Taken together, these findings highlight the interconnected nature of chronotype, SJL, sleep, PA, psychological factors, eating behavior, and meal timing. Rather than acting as independent risk factors, these components may interact bidirectionally within a broader behavioral and circadian framework, potentially contributing to both obesity and adverse mental health outcomes. This integrated perspective emphasizes the importance of considering circadian, behavioral, metabolic, and psychological dimensions together when examining the complex relationships among obesity, sleep, and mental health.

Meal timing and regularity represent additional behavioral dimensions linking circadian regulation to metabolic health. Delayed or irregular food intake, particularly when a substantial proportion of daily energy intake occurs later in the day or close to the habitual sleep period, has been associated with greater adiposity and less favorable metabolic outcomes, whereas earlier and more circadian-aligned eating patterns may be associated with metabolic benefits (Peters et al., 2024; Liu et al., 2024).

These relationships may be particularly relevant in individuals with mood disorders, in whom changes in sleep–wake timing, emotional regulation, PA, and eating behavior may interact with circadian disruption. Evidence suggests that psychological distress may be accompanied by changes in food choice, diet quality, degree of food processing, and eating patterns, while changes in the amount of food consumed appear more heterogeneous. Repeated emotional eating may also contribute to less stable eating patterns, although its long-term physiological consequences remain uncertain (Fu et al., 2026). From a broader perspective, the interplay among meal timing, eating behavior, circadian disruption, and psychological health appears complex and potentially bidirectional, supporting an integrated view of chrononutrition as part of the relationship between circadian and mental health (Cuaranta, 2026).

Multiple risk factors are shared between obesity and mood disorders, including genetic susceptibility, chronic stress exposure, physical inactivity, unhealthy dietary patterns, sleep deprivation, hormonal alterations, systemic inflammation, the use of certain medications, and psychosocial factors related to weight stigma (Gomes et al., 2019; Park et al., 2025). Another relevant aspect is the greater vulnerability observed among women. Although depressive disorders also affect men, epidemiological data consistently indicate a higher prevalence among women (Aviles Gonzalez et al., 2025). This increased female vulnerability appears to result from the interaction between hormonal factors, greater exposure to weight-related stigma, and the higher lifetime prevalence of depressive disorders (Silva et al., 2020; Alberti et al., 2024). However, recent studies suggest that these sex differences tend to diminish in individuals with severe obesity, in whom metabolic and inflammatory disturbances become the primary drivers of disease burden (Selman et al., 2025).

From a biological perspective, multiple mechanisms appear to underlie this association. In obesity, the inflammatory mediators can cross or signal through the blood–brain barrier, promoting neuroinflammation (Jantaratnotai et al., 2017), alterations in serotonergic and dopaminergic neurotransmission (Patist et al., 2018), and impaired neuroplasticity and reduced hippocampal neurogenesis (Selman et al., 2025), all of which have been implicated in the pathophysiology of depression.

Another key component of this interaction involves dysregulation of the hypothalamic–pituitary–adrenal (HPA) axis. Chronic exposure to psychological stress leads to persistent activation of this system, resulting in elevated circulating cortisol levels. Excess cortisol promotes visceral fat accumulation, insulin resistance, impaired glucose metabolism, and increased appetite, particularly for highly palatable foods rich in fat and sugar (Abraham et al., 2013). Simultaneously, hypercortisolemia exerts deleterious effects on brain regions involved in emotional regulation, including the hippocampus, prefrontal cortex, and amygdala (Schellekens et al., 2012; Bremner et al., 2020), thereby contributing to the onset and progression of depressive and anxiety disorders (Patist et al., 2018).

In addition to inflammation and neuroendocrine dysfunction, hormones produced by adipose tissue actively mediate communication between energy metabolism and brain function. Leptin, traditionally recognized for its role in regulating satiety, also exerts neuroprotective effects by modulating synaptic plasticity, memory, and emotional behavior (Harvey, 2007; Zarouna et al., 2015). However, individuals with obesity frequently develop leptin resistance, in turn reducing its central effects on reward pathways and mood regulation (Zarouna et al., 2015). Conversely, ghrelin, an orexigenic hormone produced primarily by the stomach, plays a central role in the stress response and food motivation (Zou et al., 2019), as well as in the modulation of dopaminergic reward pathways (Volkow et al., 2011; Zou et al., 2019), representing another important biological link between obesity and mood disorders.

In recent years, sleep quality has emerged as a central component in the understanding of obesity and its associated comorbidities. Insufficient or fragmented sleep can induce metabolic, hormonal, and behavioral alterations that contribute to both obesity and emotional disturbances. Prospective studies have demonstrated that short sleep duration is associated with an increased risk of weight gain, central obesity, and metabolic syndrome (Kohanmoo et al., 2024; Lange et al., 2024). Chronic sleep deprivation can also increase cortisol secretion and reduce insulin sensitivity (Chattu et al., 2018), while alterations in leptin and ghrelin levels and enhanced hedonic hunger may promote increased food intake (Rodrigues et al., 2021; Lange et al., 2024). Sleep loss may further impair inhibitory control mediated by the prefrontal cortex, potentially contributing to emotional and binge eating (Chattu et al., 2018; Rodrigues et al., 2021).

As previously described, the relationship between obesity and sleep disorders is clearly multidirectional (Figorilli et al., 2025). Excess adiposity substantially increases the risk of OSA, obesity hypoventilation, reduced sleep efficiency, and sleep fragmentation (Araghi et al., 2013b; Figorilli et al., 2025). In turn, these sleep disturbances can promote intermittent hypoxia, sympathetic nervous system activation, systemic inflammation, and neuroendocrine dysregulation (Genario et al., 2023; Figorilli et al., 2025), potentially exacerbating weight gain and symptoms of anxiety and depressive disorders (Rodrigues et al., 2021; Genario et al., 2023). Recent reviews describe this interaction as a pathophysiological cycle in which obesity, inadequate sleep, and mood disturbances may continuously reinforce one another over time (Rodrigues et al., 2021; Figorilli et al., 2025).

Beyond biological mechanisms, psychosocial factors also play a critical role. Weight-related stigma, social discrimination, low self-esteem, social isolation, and body dissatisfaction substantially increase vulnerability to depressive and anxiety disorders. These factors may trigger maladaptive coping strategies, including emotional eating, physical inactivity, and reduced treatment adherence, thus perpetuating weight gain and exacerbating psychological distress (Raman et al., 2013; Annesi and Johnson, 2021).

Taken together, contemporary models propose that obesity should be understood as a systemic condition involving tightly interconnected metabolic, neuroendocrine, immunological, behavioral, and emotional alterations. The clinical model of obesity maintenance suggests that depressed mood, anxiety, emotional dysregulation, cognitive dysfunction, limited health literacy, habitual behavioral patterns, and sleep disorders interact continuously, consequently promoting the persistence of obesity throughout the lifespan (Raman et al., 2013).

More recently, intervention studies have demonstrated that therapeutic strategies focused exclusively on weight reduction may be insufficient when emotional factors remain unaddressed. Multicomponent interventions combining PE, sleep quality improvement, nutritional counseling, and psychological treatment have shown concurrent benefits for body composition, depressive symptoms, anxiety, quality of life, and long-term weight loss maintenance (van den Hoek et al., 2023; Clavero-Jimeno et al., 2025; Vosadi et al., 2025). These findings underscore the need for a multidisciplinary approach that integrates metabolic health and mental health within a comprehensive model of obesity care. Advancing our understanding of these shared mechanisms offers a major opportunity to develop more effective, personalized, and sustainable interventions that improve not only body weight management but also mental health, sleep quality, and overall quality of life for individuals with obesity.

## 2 Discussion

**Figure 4:**
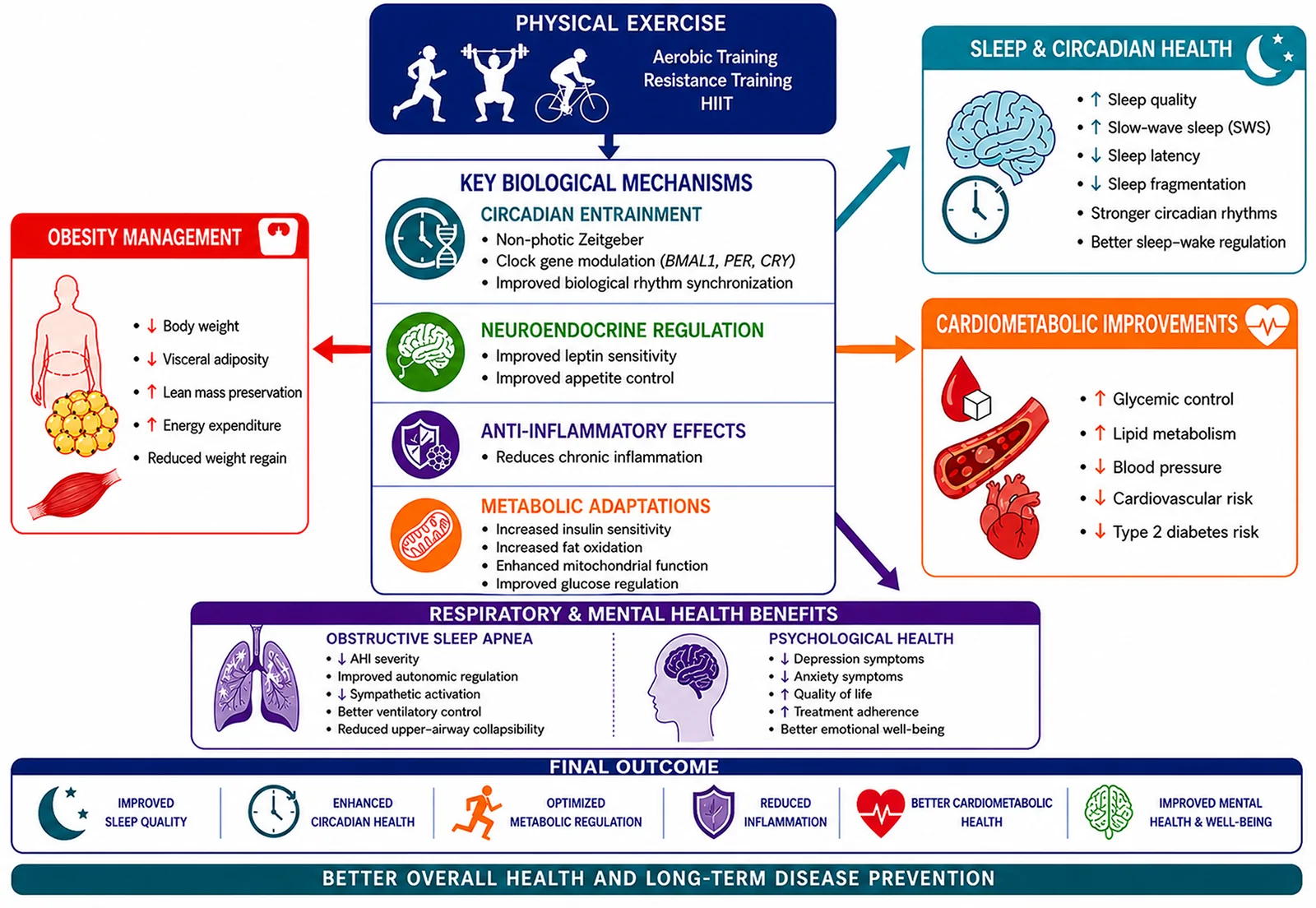
Physical exercise modulates multiple interconnected pathways linking obesity, sleep, and circadian regulation, contributing to improvements in metabolic, cardiovascular, respiratory, and mental health. HIIT, high-intensity interval training; AHI, apnea-hypopnea index.

The present review reinforces that obesity, sleep disturbances, and circadian disruption should not be viewed as isolated disorders but rather as interconnected manifestations that mutually reinforce one another through shared metabolic, neuroendocrine, inflammatory, autonomic, and behavioral pathways. Within this context, PE emerges as a valuable non-pharmacological intervention capable of simultaneously targeting multiple biological systems (Hawley et al., 2014; Oppert et al., 2021). Beyond its well-established effects on body composition and cardiometabolic health (Oppert et al., 2021; Jakicic et al., 2024), PE modulates appetite regulation (Razi et al., 2025), inflammatory signaling (Wang et al., 2022), circadian synchronization (Gabriel and Zierath, 2019), sleep quality (Yang et al., 2012; Xie et al., 2024), and mental health (Pearce et al., 2022; Vosadi et al., 2025), thereby interrupting several mechanisms involved in the vicious cycle linking poor sleep, circadian misalignment, and obesity. Therefore, recognizing PE as both a behavioral intervention and a non-photic synchronizer of the circadian system broadens its clinical relevance and supports more integrated strategies for preventing and treating obesity-related comorbidities (Atkinson et al., 2007; Healy et al., 2021; Shen et al., 2023) (Figure 4).

Despite the growing consistency of evidence supporting the beneficial effects of PE on sleep, circadian rhythms, mental health, and metabolic regulation (Yang et al., 2012; Shen et al., 2023; Pearce et al., 2022; Oppert et al., 2021), important knowledge gaps remain. Questions regarding the most effective PE modality, intensity, duration, frequency, and particularly the timing of PE remain incompletely answered (Bruggisser et al., 2023; Jakicic et al., 2024; Teo et al., 2020). More importantly, accumulating evidence suggests that there is unlikely to be a single optimal PE prescription applicable to all individuals (Noone et al., 2024). Considerable interindividual variability exists in physiological and clinical responses to PE, with adaptations being influenced by both intrinsic factors, including age, sex, chronotype, genetic background, baseline physical fitness, and metabolic status, and extrinsic factors such as exercise timing, modality, intensity, sleep habits, dietary patterns, medication use, and environmental influences (Mann et al., 2014; Noone et al., 2024). Rather than implying that each of these factors should independently determine exercise prescription, this variability highlights the need to identify which individual characteristics meaningfully modify the response to specific exercise stimuli (Moda et al., 2024; Noone et al., 2024).

This concept is particularly relevant in the context of sleep and circadian biology. Although chronoexercise has emerged as a promising field (Kim et al., 2023; Shen et al., 2023), the available evidence remains insufficient to determine whether morning, afternoon, or evening PE consistently provides superior benefits for sleep or metabolic health (Bruggisser et al., 2023; Teo et al., 2020; Shen et al., 2025). Accordingly, the current evidence does not justify prescribing a universally “best” time of day for PE (Bruggisser et al., 2023). Instead, future research should determine whether specific exercise characteristics interact with chronotype, sleep characteristics, obesity severity, metabolic health, physical fitness, and individual preferences to influence treatment responses. Such evidence would provide a stronger basis for precision lifestyle medicine than simply recommending exercise according to clock time.

The potential relevance of circadian characteristics is further illustrated by chronotype and SJL, reflecting individual differences in preferred timing and misalignment between biological and socially imposed schedules, respectively (Adan et al., 2012; Wittmann et al., 2006). Both later chronotype and greater SJL have been associated with obesity and adverse metabolic outcomes (Arab et al., 2024; Zhang et al., 2022), while evening chronotypes have also been associated with lower PA and greater sedentary behavior (Sempere-Rubio et al., 2022). These associations suggest that circadian characteristics may influence not only metabolic health but also the behavioral context in which exercise is performed. However, the predominantly observational nature of this evidence limits causal interpretation, and it remains unclear whether modifying exercise timing according to chronotype or SJL improves clinical outcomes. This uncertainty is particularly important when considering the emerging field of chronoexercise (Kim et al., 2023; Shen et al., 2023). Exercise responses are influenced by the interaction between the physiological stimulus imposed by PE and the biological state in which that stimulus is received (Gabriel and Zierath, 2019; Youngstedt et al., 2019). Thus, the effects of exercise timing may reflect not only external clock time but also endogenous circadian phase, sleep-wake patterns, chronotype, and the metabolic state of the individual (Wolff and Esser, 2019; Rynders and Broussard, 2024). Future studies should therefore move beyond comparisons of fixed morning vs. evening exercise and investigate whether aligning exercise characteristics with individual circadian profiles produces clinically meaningful benefits in obesity and sleep-related outcomes.

The complexity of these interactions also helps explain why exercise interventions may produce heterogeneous outcomes across individuals (Noone et al., 2024; Mann et al., 2014). Genetic background, baseline fitness, recovery, sleep, dietary patterns, emotional state, body weight, age, and environmental circumstances may all contribute to variability in exercise responsiveness (Noone et al., 2024; Pickering and Kiely, 2019). Rather than repeating the broader principle that exercise prescriptions should simply be “individualized,” the more relevant research question is which of these characteristics can be used to predict or modify treatment response. This approach may help distinguish clinically meaningful sources of heterogeneity from factors that have limited practical relevance and could contribute to more precise exercise recommendations (Noone et al., 2024).

Long-term adherence represents another important determinant of whether these physiological benefits translate into meaningful clinical outcomes (Middleton et al., 2013; Oppert et al., 2021). Individuals with obesity may experience fatigue, daytime sleepiness, musculoskeletal discomfort, low cardiorespiratory fitness, and functional limitations, while time constraints, competing responsibilities, limited access to exercise facilities, and insufficient social support may further interfere with regular PE (Baillot et al., 2021; Deslippe et al., 2023). These factors indicate that the effectiveness of exercise in real-world settings depends not only on the biological response to a given exercise stimulus but also on the feasibility of incorporating that stimulus into everyday life. Accordingly, future research should evaluate exercise interventions using outcomes that extend beyond body weight and include adherence, functional capacity, sleep quality, metabolic health, and quality of life (Pojednic et al., 2022; Jakicic et al., 2024).

An important limitation of the current literature in this field is the marked heterogeneity across studies. Differences in participant characteristics, PE protocols, intervention duration, the complexities associated with obtaining objective sleep measures, circadian biomarkers, and clinical outcomes make direct comparisons challenging and limit the establishment of evidence-based recommendations (Oppert et al., 2021; Bruggisser et al., 2023; Xie et al., 2024). Publication bias may also contribute to the small effect sizes and high variability observed, particularly through underreporting of negative findings. At the same time, part of this heterogeneity likely reflects true biological variability in PE responsiveness rather than methodological limitations alone (Moda et al., 2024; Noone et al., 2024). This reinforces the importance of future randomized controlled trials that combine standardized methodologies with precision medicine approaches capable of identifying which PE prescriptions are most beneficial for specific clinical and behavioral phenotypes.

Ultimately, integrating PE, sleep, and circadian biology opens new opportunities for multidisciplinary interventions. Combining PE with nutritional strategies, sleep hygiene, cognitive-behavioral interventions, and chronotherapeutic approaches may produce synergistic effects capable of improving metabolic health more effectively than isolated interventions. Understanding how these lifestyle components interact may represent an important step toward personalized strategies for obesity prevention and treatment.

### 2.1 Practical implications and recommendations for clinicians

Although the metabolic, neuroendocrine, and circadian mechanisms discussed in this review support physical exercise as a relevant strategy for modulating the interaction between sleep and obesity, translating these physiological effects into clinical practice depends largely on the ability to initiate and sustain regular PA. Thus, biological efficacy should be distinguished from clinical effectiveness, as the magnitude of real-world benefits depends substantially on adherence, tolerability, and long-term maintenance. This distinction is particularly relevant in individuals with obesity, in whom sleep disturbances, fatigue, low cardiorespiratory fitness, musculoskeletal discomfort, and functional limitations may represent important barriers to regular PA participation (Baillot et al., 2021).

Exercise prescription should therefore be individualized according to baseline functional capacity, symptoms, preferences, and tolerability. Gradual progression of exercise volume and intensity, shorter sessions, adequate recovery, and the selection of lower-impact or combined aerobic and resistance modalities may facilitate initial adaptation and reduce the risk of discontinuation (Komici et al., 2026; Oppewal et al., 2026). Resistance training may be particularly relevant because of its potential contribution to the preservation or improvement of muscle mass, strength, and functional capacity during weight-management interventions. Accordingly, exercise modality should be selected not only according to energy expenditure but also according to safety, feasibility, and the likelihood of long-term adherence (Orange et al., 2020; Lopez et al., 2022).

The bidirectional relationship between sleep and PA further highlights the need for an integrated approach. Poor sleep may contribute to fatigue, daytime sleepiness, and reduced PA, while physical inactivity may limit opportunities to improve sleep and metabolic health (Kline et al., 2013). Regular PA may attenuate this cycle, given evidence of beneficial effects on sleep quality and sleep disturbances (Alnawwar et al., 2023; Mostafa et al., 2026). However, fatigue and sleepiness should not simply be interpreted as lack of motivation. Clinically relevant sleep disturbances, including sleep-related breathing disorders or insomnia, should be appropriately investigated and managed (Samdal et al., 2017; Emsellem et al., 2025). Sleep and circadian factors should also be considered when prescribing PE, particularly chronotype, SJL, and exposure to shift work, as misalignment between individual circadian preferences, sleep–wake patterns, and socially imposed schedules may interfere with exercise engagement and recovery.

Adherence is also influenced by behavioral, social, and environmental factors, including lack of time, limited access to facilities, financial and transportation barriers, work and family responsibilities, and limited social support (Middleton et al., 2013). Home-based, remotely delivered, shorter, or group-based programs may help overcome some of these barriers, while behavioral strategies such as realistic goal setting, self-monitoring, feedback, and motivational interviewing may facilitate sustained engagement (Sullivan and Lachman, 2017). Temporary interruptions should therefore be viewed as opportunities to reassess barriers and adapt the intervention rather than necessarily indicating therapeutic failure.

The timing of exercise may also interact with circadian rhythms, sleep, and metabolism. However, current evidence does not support a universally optimal time of day, and exercise timing should be individualized according to chronotype, daily routines, sleep patterns, preferences, and tolerability (Bennett and Sato, 2023; Rynders and Broussard, 2024; Hesketh, 2026). Similarly, treatment response should not be evaluated exclusively according to changes in body weight. Improvements in cardiorespiratory fitness, muscular strength, sleep quality, functional capacity, energy levels, and metabolic or mental health may represent clinically meaningful benefits even in the absence of substantial weight loss. Clinicians should also consider regularity in daily behaviors, including PA and food intake, as potential behavioral *zeitgeber* that may support circadian alignment and contribute to sleep and metabolic health (Healy et al., 2021; Lewis et al., 2020; Clemente-Suárez et al., 2026).

Digital health technologies and artificial intelligence (AI) may further support the implementation of individualized exercise and sleep recommendations in clinical practice. Wearable devices and mobile applications can facilitate the monitoring of PA, sleep patterns, exercise timing, and adherence in real-world settings, while AI-based approaches may integrate these data with individual characteristics to support more personalized recommendations. However, these technologies should complement rather than replace clinical judgment, as their usefulness depends on the quality and representativeness of the underlying data.

Overall, PE should be regarded as a progressive and individualized intervention that considers functional capacity, sleep, circadian characteristics, behavioral factors, and environmental barriers. Such an integrated approach may improve adherence and help translate the physiological benefits of PE into sustainable improvements in metabolic, sleep, and circadian health, particularly in individuals with obesity and sleep disturbances.

## 3 Conclusion

Physical exercise represents a key non-pharmacological strategy for the prevention and management of obesity, with benefits extending to metabolic health, sleep, and circadian regulation. The evidence discussed in this review reinforces that its effects depend not only on exercise type, intensity, and duration but may also be influenced by exercise timing and individual characteristics, including chronotype, sleep patterns, SJL, metabolic status, and behavioral context. In this perspective, chronoexercise expands the traditional approach to exercise prescription by incorporating its temporal dimension and interactions with other important circadian synchronizers, such as the light–dark cycle and meal timing. Although longitudinal studies and clinical trials are still needed to establish causal relationships and define more precise timing strategies, integrating PE, sleep, and circadian biology offers a promising framework for more individualized interventions. Further advances in this field may help improve adherence and maximize the sustainable benefits of PE for the prevention and management of obesity and its associated metabolic and sleep disturbances.
